# Functional Properties of *Campomanesia xanthocarpa* Infusions: Phenolic Profile, Digestive Stability, Enzyme Inhibition, and Glycemic Effects

**DOI:** 10.3390/foods14142469

**Published:** 2025-07-14

**Authors:** Cristiane Maria Chitolina Tremea, Vanessa Ruana Ferreira da Silva, Larissa Cunico, Vinícius Gottardo Boff, Carolina Turnes Pasini Deolindo, Aleksandro Shafer da Silva, Aniela Pinto Kempka

**Affiliations:** 1 Postgraduate Program in Food Science and Technology, Department of Food Engineering and Chemical Engineering, Santa Catarina State University, Pinhalzinho 89870-000, SC, Brazil; cristremea@gmail.com; 2 Multicentric Postgraduate Program in Biochemistry and Molecular Biology, Department of Animal Production and Food Science, Santa Catarina State University, Lages 88520-000, SC, Brazil; vanessa.silva@udesc.br (V.R.F.d.S.); aleksandro.silva@udesc.br (A.S.d.S.); 3 Department of Animal Science, Santa Catarina State University, Chapecó 89815-630, SC, Brazil; larissa.cunico1441@edu.udesc.br (L.C.); viniciusboff123@gmail.com (V.G.B.); 4 Federal Agricultural Defense Laboratory, Ministry of Agriculture, Livestock, and Food Supply, São José 88102-600, SC, Brazil; carolinaturnes.pd@gmail.com; 5 Department of Food Science and Technology, Federal University of Santa Catarina, Florianópolis 88040-900, SC, Brazil; 6 Department of Food Engineering and Chemical Engineering, Santa Catarina State University, Pinhalzinho 89870-000, SC, Brazil

**Keywords:** antioxidant activity, bioaccessibility, functional food, polyphenols, α-amylase inhibition, fructosamine

## Abstract

This study investigated the functional potential of *Campomanesia xanthocarpa* leaf and fruit infusions through phytochemical profiling, simulated gastrointestinal digestion, enzyme inhibition assays, and in vivo evaluation of glycemic markers. Leaf infusions exhibited a more diverse phenolic profile, higher total phenolic content, and greater antioxidant capacity compared to fruit infusions. Simulated digestion confirmed the bioaccessibility of key phenolic compounds, particularly glycosylated flavonoids such as quercetin-3-glucoside and kaempferol derivatives, with leaf extracts showing superior gastrointestinal stability. In vitro assays revealed a strong inhibitory activity of leaf infusions against α-amylase and β-glucosidase. In a 32-day trial with healthy dogs, the consumption of biscuits enriched with leaf infusion did not alter fasting glucose or amylase levels but resulted in a significant treatment × time interaction for serum fructosamine, indicating a delayed modulation of glycemic control, potentially associated with antioxidant or anti-glycation activity. These findings highlight the potential of *C. xanthocarpa* leaves as a functional ingredient in foods aimed at supporting glycemic regulation and metabolic health.

## 1. Introduction

*Campomanesia xanthocarpa* O. Berg, commonly known as guabiroba, is a native species of the Myrtaceae family widely distributed across the Atlantic Forest and Cerrado biomes, as well as in parts of Argentina and Paraguay. With small, round, yellow-orange fruits when ripe, opposite leaves, and white flowers, this plant has been traditionally used for both medicinal and dietary purposes, including the treatment of liver diseases, digestive disorders, inflammation, and diabetes [1]. Phytochemical investigations have demonstrated a broad spectrum of bioactive constituents, particularly polyphenols, that contribute to the pharmacological potential of the species. Several published studies have shown that extracts from its leaves and fruits exhibit anti-inflammatory, hepatoprotective, cardioprotective, antioxidant, and antihyperglycemic properties [2,3,4,5]. Notably, Arcari et al. [2] reported a rich phenolic profile in guabiroba fruits, including flavonols and phenolic acids, while Sant’Anna et al. [3] and Catelan et al. [5] demonstrated hypotensive and photoprotective effects from different *Campomanesia* species, underscoring their functional versatility.

These properties are largely attributed to the presence of phenolic compounds such as gallic acid, chlorogenic acid, quercetin, and myricetin, which are known for their ability to neutralize reactive oxygen species and reduce cellular oxidative stress [6]. This mechanism is particularly relevant due to the established link between oxidative stress and chronic metabolic diseases such as type 2 diabetes, cardiovascular diseases, and systemic inflammatory damage. In addition, fruits from the Myrtaceae family have demonstrated significant inhibitory activity against digestive enzymes such as α-glucosidase and α-amylase, thereby affecting starch breakdown and carbohydrate absorption in the small intestine. Inhibiting these enzymes is considered an effective strategy for postprandial glycemic control and is viewed as a promising approach for managing hyperglycemia in patients with type 2 diabetes [7].

Several clinical and experimental studies indicate that dietary polyphenols exert hypoglycemic effects by acting at multiple stages of glucose metabolism, including the modulation of intestinal glucose transporters (SGLT1 and SGLT2), inhibiting hepatic gluconeogenesis, stimulating peripheral glucose uptake, and preserving pancreatic β-cell function [8,9,10]. Additionally, polyphenols have been shown to influence intracellular pathways such as AMPK and PI3K/Akt, thereby affecting insulin sensitivity and glycemic homeostasis [11,12].

Nonetheless, their functional efficacy is highly dependent on their stability during digestion and their bioaccessibility—the fraction released from the food matrix that becomes available for intestinal absorption. These factors are significantly modulated by interactions with the food matrix, which can either hinder or enhance polyphenol release. Macronutrients such as proteins, fibers, and lipids may bind polyphenols, leading to the formation of soluble or insoluble complexes [13,14]. Such interactions, whether covalent or non-covalent, can limit solubility and recognition by intestinal transporters, yet may also play a protective role, delaying degradation and enabling distal release in the gastrointestinal tract [15,16,17]. Once absorbed or metabolized, polyphenols may undergo phase I and II enzymatic modifications and microbial transformation in the colon, yielding metabolites with distinct structures and biological activities. These metabolic routes ultimately shape their systemic effects and health-promoting potential [17,18]. Although *C. xanthocarpa* has been increasingly studied, limited data are available regarding the digestive fate of its phenolics, especially under conditions simulating realistic food consumption. Furthermore, most studies have employed extractive approaches that do not reflect practical dietary habits.

Therefore, this study aimed to evaluate the phenolic profile of *C. xanthocarpa* leaf and fruit infusions prepared at different extraction times, investigate their stability and bioaccessibility after simulated in vitro digestion, assess their inhibitory potential against amylolytic enzymes (α-amylase and β-glucosidase), and examine the physiological effects of consuming dry biscuits enriched with leaf infusion on glycemic markers (glucose, amylase, and fructosamine) in an animal model.

## 2. Material and Methods

### 2.1. Plant Material and Chemicals

The leaves and fruits of *C. xanthocarpa* were harvested in January 2023 (summer in Brazil) from a tree located in a rural area in the municipality of Nova Erechim, western Santa Catarina State (Latitude: 26°54′09″ S, Longitude: 52°54′21″ W). No fertilization or pesticide treatments were applied to the plant. A voucher specimen was deposited in the LUSC Herbarium of Santa Catarina State University (voucher no. LUSC 10711). The plant material underwent a manual pre-selection process, during which damaged specimens, those with altered coloration, and those showing signs of pathology were discarded, ensuring sample uniformity. Fruits were selected at full physiological maturity, identified by a uniform yellow-orange coloration, characteristic aromatic profile, and ease of detachment from the branch. Leaves were collected from fully expanded, mature specimens with consistent green coloration and no signs of senescence, ensuring a uniform phenological stage. These maturity standards were applied systematically to minimize variability in the polyphenol profile. Leaves were washed with distilled water and dried in a circulating air oven at 40 ± 5 °C until reaching a constant weight. Fruits were frozen at −83 °C and subsequently lyophilized at −60 °C and 0.05 mTorr (Benchtop Freeze Dryers, Model ilShin, lShin BioBase Co., Ltd., Dongducheon-si, South Korea) for 72 h. Seeds were removed after freeze-drying. Following drying, all plant fractions were ground and sieved through an 8-mesh sieve. Fruit pulp and peel were processed together, while leaves were ground and sieved separately. All chemicals used in this study were of analytical grade. Key reagents included Folin–Ciocalteu reagent, gallic acid, and sodium carbonate, acquired from Sigma-Aldrich^®^ (Cotia, SP, Brazil). For the amylase assay, Amylase Assay Buffer, Amylase Substrate Mix, Amylase Positive Control, and Nitrophenol Standard were employed. For the β-glucosidase assay, Assay Buffer, β-NPG Substrate, and Calibrator were used. For antioxidant analysis, Reagent A, Reagent B, and a 50 mM Trolox Standard were used—all obtained from Sigma-Aldrich^®^ (St. Louis, MO, USA). Analytical standards for phenolic compounds were acquired from Sigma-Aldrich^®^, Supelco^®^, Dr. Ehrenstorfer™, and Toronto Research Chemicals. A detailed list of these standards is provided in Section 2.4. This research is registered with SisGen (National System for the Management of Genetic Heritage and Associated Traditional Knowledge) under registration code A926504.

### 2.2. Obtaining the Infusions

Sample preparation involved infusing 250 mL of boiling distilled water into 1 g of plant material from each fraction (leaves and seedless fruits). For each fraction, three infusions were prepared, with steeping times of 5, 10, and 15 min, respectively, totaling six infusions. After the steeping period, the samples were centrifuged at 3000× *g* for 5 min. The resulting supernatants were frozen at −83 °C and subsequently lyophilized at −60 °C and 0.05 mTorr (Benchtop Freeze Dryers, Model ilShin, lShin BioBase Co., Ltd., Dongducheon-si, South Korea) for 72 h [19,20]. The lyophilized yield for each infusion ranged from 15 to 22 mg per 250 mL, corresponding to concentrations of approximately 60–88 μg/mL. These yields were used to reconstitute samples at equivalent concentrations for further analyses. Samples were labeled as follows: L5—leaf infusion steeped for 5 min; L10—leaf infusion steeped for 10 min; L15—leaf infusion steeped for 15 min; F5—fruit infusion steeped for 5 min; F10—fruit infusion steeped for 10 min; F15—fruit infusion steeped for 15 min.

### 2.3. In Vitro Digestion

The in vitro digestion protocol was adapted from previously validated methodologies that simulate human gastrointestinal conditions, as described by Jagadeesan et al. [21] and Kautzmann et al. [22]. Lyophilized samples from each infusion were reconstituted in distilled water to match the original infusion concentration (60–88 μg/mL) before digestion. Undigested samples (referred to as L5, L10, L15 for leaf infusions and F5, F10, F15 for fruit infusions) were obtained by reconstituting the lyophilized material in distilled water at the same concentration used for digestion. These samples were vortexed for 30 s, filtered through 0.45 µm membranes, and immediately stored in amber vials at −20 °C until further use in phenolic, antioxidant, and enzyme inhibition assays. For the gastric phase, 50 mL of each infusion (leaf and fruit) were mixed with 50 mL of simulated gastric fluid (containing 10 g/L of pepsin, a gastric enzyme) prepared with 16.4 mL of HCl in 1000 mL of distilled water, with the pH adjusted to 1.3 ± 0.1 using 0.1 M HCl, and incubated at 37 °C for 60 min. Following incubation, the solution was cooled in an ice bath for 10 min to halt enzymatic activity. From the final volume (100 mL), 50 mL was set aside for the intestinal digestion phase, while the remaining 50 mL was retained for analysis of the gastric digestion stage. For the intestinal phase, 50 mL of the gastric digest was combined with 15 mL of simulated intestinal fluid, which included pancreatin (2% *w*/*v*) as the enzymatic component to simulate small intestinal conditions. This solution was prepared by mixing 500 mL of the pancreatin solution with an equal volume of electrolyte solution (containing 6.8 g of KH_2_PO_4_), and the pH was adjusted to 6.8 ± 0.1 using 4% NaOH. The mixture was incubated at 37 °C for 120 min under continuous stirring at 100 rpm. After incubation, pancreatin activity was halted by cooling the mixture in an ice bath for 10 min. The final digests were stored in amber flasks at −20 °C for subsequent analysis.

### 2.4. Identification and Quantification of Individual Phenolic Compound by LIQUID Chromatography-Electrospray Ionization-Tandem Mass Spectrometry (LC-ESI-MS/MS)

To identify and quantify individual phenolic compounds, liquid chromatography coupled with electrospray ionization tandem mass spectrometry (LC-ESI-MS/MS) was employed. Analyses were conducted using a liquid chromatograph (Agilent 1290 Series, Agilent Technologies, Wilmington, NC, USA) coupled to a hybrid quadrupole-linear ion trap mass spectrometer (QTRAP^®^ 5500, Sciex, Foster City, CA, USA) equipped with an electrospray ionization (ESI) source. Phenolic compounds were identified and quantified based on their retention times, precursor ions, major fragments (quantifier ions), and secondary fragments (qualifier ions), in comparison with commercial analytical standards. Chromatographic separation was performed on an Agilent Zorbax Eclipse Plus C18 column (3.0 × 100 mm, 3.5 µm) using a binary mobile phase consisting of water (A) and methanol (B), both containing 0.1% formic acid. The gradient program was as follows: 0–3 min, 2% B; 3–10 min, 20% B; 10–11 min, 90% B; 11–13 min, 2% B. The flow rate was set at 0.3 mL/min, with an equilibration time of 2 min between runs. The injection volume was 5 µL. Data acquisition and processing were performed using Analyst 1.6.2 and MultiQuant 3.0.5373.0 software (Sciex, Framingham, MA, USA), respectively. A total of 18 phenolic compound standards were used for compound identification and quantification. These standards were obtained from Sigma-Aldrich^®^ (Cotia, SP, Brazil), Supelco^®^ (Cotia, SP, Brazil), Dr. Ehrenstorfer™ (Teddington, Middx, UK), and Toronto Research Chemicals (Toronto, ON, Canada), as follows: benzoic acid (CAS 100-90-4, product code DRE-C10265900, purity >99%, Dr. Ehrenstorfer™); chlorogenic acid (CAS 327-97-9, DRE-C11415750, >96%, Dr. Ehrenstorfer™); ferulic acid (CAS 537-98-4, DRE-C13644100, >98%, Dr. Ehrenstorfer™); catechin (CAS 7295-85-4, DRE-C11059100, >93%, Dr. Ehrenstorfer™); coumarin (CAS 91-64-5, DRE-C11735000, >99%, Dr. Ehrenstorfer™); epicatechin (CAS 490-46-0, DRE-C13174690, >98%, Dr. Ehrenstorfer™); hesperidin (CAS 520-26-3, DRE-C14140000, >86%, Dr. Ehrenstorfer™); kaempferol-3-O-β-rutinoside (CAS 64820-99-1, product code R701800, >95%, Toronto Research Chemicals); kaempferol (CAS 520-18-3, DRE-C14502000, >94%, Dr. Ehrenstorfer™); myricetin (CAS 529-44-2, product code M6760, ≥96%, Sigma-Aldrich^®^); quercetin-3-rhamninoside (CAS 20229-56-5, product code Q8259, ≥95%, Sigma-Aldrich^®^); quercetin-3-glucoside-7-glucuronide (CAS 33595-51-0, product code Q394000, ≥95%, Toronto Research Chemicals); 4-aminobenzoic acid (CAS 150-13-0, DRE-C10171400, >98%, Dr. Ehrenstorfer™); apigenin (CAS 520-36-5, DRE-C10290600, >97%, Dr. Ehrenstorfer™); galangin (CAS 548-83-4, DRE-C13997000, >98%, Dr. Ehrenstorfer™); pinocembrin (CAS 480-39-7, product code P5239, ≥95%, Supelco^®^); syringaldehyde (CAS 134-96-3, DRE-C17080000, ≥99%, Dr. Ehrenstorfer™); and syringic acid (CAS 530-57-4, DRE-C17081000, ≥95%, Dr. Ehrenstorfer™).

### 2.5. Bioaccessibility

The bioaccessibility of phenolic compounds was determined by assessing the effect of in vitro digestion on the composition of individual phenolic constituents in the infusions. The bioaccessibility index (*BI*) was calculated according to Equation (1), where *A* represents the concentration of each individual phenolic compound after the intestinal digestion phase and *B* corresponds to the concentration of the same compound prior to in vitro digestion (undigested sample) [22,23].

(1) $$BI\;\left(\%\right)\;=\;100\;\times \;\frac{A}{B}$$

### 2.6. Total Phenolic Content (TPC) and Total Antioxidant Capacity (TAC)

Prior to *TPC* and *TAC* analysis, all digested samples (gastric and intestinal) were thawed, centrifuged at 5000× *g* for 10 min at 4 °C to remove insoluble material, and filtered through 0.45 μm syringe filters. This step ensured the removal of particulate matter and residual enzymatic proteins that could interfere with the colorimetric reactions. Only the resulting clear supernatants were used for the assays.

The total phenolic content (*TPC*) was determined using the Folin–Ciocalteu colorimetric method. For each infusion, 0.1 mL of sample was mixed with 6 mL of distilled water, 0.5 mL of Folin–Ciocalteu reagent, and 2 mL of 20% Na_2_CO_3_, followed by manual stirring for 30 s. The reaction mixture was incubated in the dark for 2 h. After incubation, absorbance was measured at 750 nm using a spectrophotometer (DR 3900, Hach, Düsseldorf, Germany) against a blank solution. The standard curve was constructed using gallic acid solutions. The *TPC*s of each infusion was expressed as milligrams of gallic acid equivalent (mg GAE) per gram of dry sample, based on the equation of the calibration curve. Results were presented as mean ± standard deviation of replicate analyses [22,23,24].

The total antioxidant capacity (*TAC*) assay was performed in a 96-well plate using a Varioskan LUX Plate Reader (ThermoFisher Scientific, Waltham, MA, USA), following the manufacturer’s instructions as provided in the Antioxidant Test Kit (catalog number MAK334, Sigma-Aldrich, St. Louis, MO, USA). The Master Reaction Mix (MRX-a) was prepared by mixing 100 μL of Reagent A and 8 μL of Reagent B, which were then added to each test well. A standard curve was prepared using Trolox concentrations of 0, 300, 600, and 1000 μM. In the plate, 20 μL of each standard and 20 μL of sample were pipetted into separate wells, followed by the addition of 100 μL of MRX-a to all wells. The plate was gently shaken by hand and incubated at room temperature for 10 min. Absorbance was then measured at 570 nm. *TAC* values were calculated according to Equation (2), where (*A_570_*) sample is the absorbance of the sample, (*A_570_*) blank is the absorbance of the blank, *n* is the sample dilution factor, and Slope refers to the slope of the Trolox standard curve, obtained by plotting absorbance at 570 nm against Trolox concentrations.

(2) $$TAC\left(µM\right)\;=\frac{\left(A570\right)sample\;-\;\left(A570\right)blank\;\times \;n}{Slope\;\left(µM^{-1}\right)}$$

### 2.7. In Vitro Inhibition of α-Amylase

The α-amylase inhibition assay was performed using a 96-well plate on a Varioskan LUX Plate Reader, following the manufacturer’s instructions provided in the α-Amylase Inhibitor Screening Kit (Catalog Number MAK009, Sigma-Aldrich, St. Louis, USA). The Master Reaction Mixture (MRX-α) was prepared by mixing 50 μL of amylase assay buffer with 50 μL of amylase substrate. In separate wells, the nitrophenol standard, positive control, and test samples (leaf or fruit infusions) were added. Undigested infusions were used in this assay, as salivary α-amylase interacts directly with the native compounds present in the unaltered samples. To each well, 100 μL of MRX-α was added, and the contents were gently mixed. After 3 min, the initial absorbance (*A*_405_(initial)) was recorded at 405 nm. The plate was then incubated at 25 °C, and absorbance was measured every 5 min over a 1 h period. The change in absorbance (Δ*A*_405_) was calculated using Equation (3):

(3) $$∆A_{405}=A_{405\left(final\right)}-A_{405\left(initial\right)\;}$$

### 2.8. In Vitro Inhibition of β-Glucosidase

The β-glucosidase inhibition assay was carried out in a 96-well plate using the Varioskan LUX Plate Reader, according to the manufacturer’s instructions provided in the β-Glucosidase Inhibitor Screening Kit (Catalog Number MAK129, Sigma-Aldrich, St. Louis, MO, USA). To initiate the procedure, 20 μL of distilled water was added to two wells, with 200 μL of distilled water in one well and 200 μL of calibrator in the other. The Master Reaction Mixture (MRX-β) was prepared by mixing 200 μL of test sample with 8 μL of β-NPG substrate, yielding a final concentration of 1 mM β-NPG. Separately, the nitrophenol standard, positive control, and test samples (leaf or fruit infusions) obtained after gastric digestion were added to the appropriate wells. These samples were selected because β-glucosidase activity occurs in the small intestine, and therefore only compounds surviving gastric digestion are relevant. Next, 200 μL of MRX-β was added to each test well, and the initial absorbance (*A*_405_(initial)) was recorded at 405 nm. The plate was incubated for 20 min at room temperature or 37 °C, and the final absorbance was measured immediately after incubation. The percentage inhibition of β-glucosidase was calculated based on Equation (4):

(4) $$\%\;inhibition=\frac{\left(A405\right)final\;-\;\left(A405\right)initial\;\times \;250units/L}{\left(A405\right)calibrador\;-\;\left(A504\right)water}$$

### 2.9. Preparation of Dry Baked Biscuits with Added Leaf Extract

Dry baked biscuits were produced using commercially available ingredients. Two formulations were developed: a control formulation and a treatment formulation containing 0.4% *C. xanthocarpa* leaf extract (L15). The composition of both formulations is presented in Table 1 as percentages (% *w*/*w*), and a representative image is included to illustrate the visual characteristics of the biscuits. To prevent contamination, the workbench was sanitized using 70% ethanol and disposable paper towels prior to preparation. Initially, the dry ingredients were crushed and mixed. Potassium sorbate and bacon flavoring were then added, followed by homogenization of the mixture. In parallel, distilled water was heated to dissolve the gelatin. For the treatment formulation, gelatin was dissolved directly in the *C. xanthocarpa* extract to ensure uniform distribution of the bioactive compound. The gelatin solution was gradually incorporated into the dry mixture until a soft, homogeneous dough was obtained. The dough was shaped using bone-shaped molds, and individual units were weighed to ensure consistency in weight. The biscuits were baked at 180 °C for 30 min, then cooled to room temperature and individually packaged in zip-lock plastic bags for storage and subsequent analyses [25,26].

### 2.10. In Vivo Evaluation of Glycemic Markers

The in vivo evaluation of glycemic markers was conducted at the Experimental Farm of Santa Catarina State University (Guatambú, Brazil) using ten adult male Beagle dogs, aged 7 years, randomly assigned to two groups (*n* = 5 per group). The control group (animals 1 to 5) received dry baked biscuits without *C. xanthocarpa* leaf infusion, while the treatment group (animals 6 to 10) received biscuits formulated with the infusion. The intervention period lasted 28 days, during which two biscuits per day were administered to each animal in the afternoon. Blood samples were collected weekly by jugular vein puncture using vacuum tubes—both with and without clot activator—to obtain serum and plasma, respectively. Samples were centrifuged at 7000 rpm for 10 min, and the supernatant was transferred to 1.5 mL labeled microtubes, then stored at −20 °C until analysis [27]. Biochemical parameters assessed included fasting glucose, fructosamine, and serum amylase, measured using standard clinical chemistry methods. The study was approved by the Ethics Committee on Animal Use (CEUA/UDESC) under protocol number 3728250923, ID 001833.

### 2.11. Statistical Analysis

Statistical analyses were performed using R Statistical Software (v.3.5.1; ANOVAIREVA, R Core Team, 2016) and Microsoft^®^ Excel^®^ 2019 MSO. The analysis included analysis of variance (ANOVA), and differences between means were evaluated using Tukey’s test, with a 95% confidence level (*p* < 0.05). All results were based on three independent experimental replicates.

For the in vivo data, a descriptive analysis was first conducted, including measures of central tendency (median) and data dispersion (range, defined as the interval between the minimum and maximum values). Subsequently, all variables were subjected to the Shapiro–Wilk W-test for normality. Variables with non-normal distribution were transformed using appropriate algorithms to ensure normality. After normalization, skewness, kurtosis, and homogeneity of variance were evaluated using the Levene test, and linearity was assessed via linear regression. Statistical modeling was conducted using the PROC MIXED procedure (SAS Institute Inc., Cary, NC, USA; version 9.4), applying the Satterthwaite approximation to determine the denominator degrees of freedom for testing fixed effects. Baseline values (day 0) for each variable were included as covariates in the model. As only a single post-treatment collection was conducted following additive consumption, treatment effects were not reported separately, being instead incorporated into the treatment × day interaction. Mean comparisons were performed using PDIFF (Student’s *t*-test), and results were expressed as least squares means (LSMEANS) with corresponding standard errors. Statistical significance was set at *p* ≤ 0.05 [27].

## 3. Results and Discussion

### 3.1. Phenolic Composition of Infusions of C. xanthocarpa Before and After In Vitro Digestion

**Figure 1:**
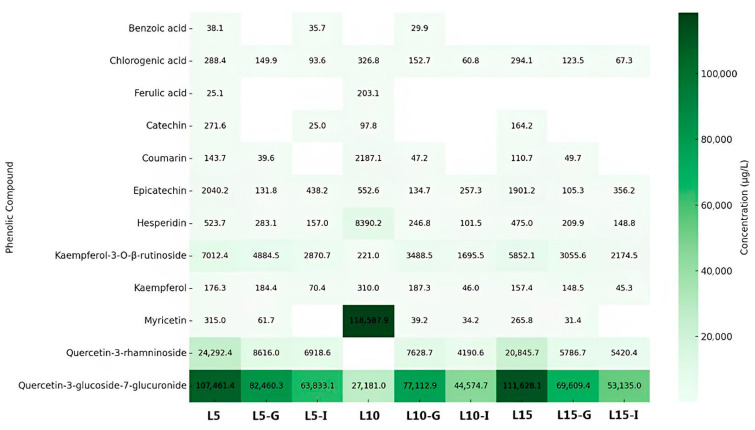
Concentrations of phenolic compounds detected in leaf infusions with steeping times of 5, 10, and 15 min, and their respective gastric and intestinal in vitro digestions. Note: The samples were labeled as follows: L5, L10, and L15 represent leaf infusions with resting times of 5, 10, and 15 min, respectively; L5-G, L10-G, and L15-G correspond to leaf infusions with resting times of 5, 10, and 15 min, respectively, subjected to gastric digestion, while L5-I, L10-I, and L15-I refer to those subjected to intestinal digestion.

**Figure 2:**
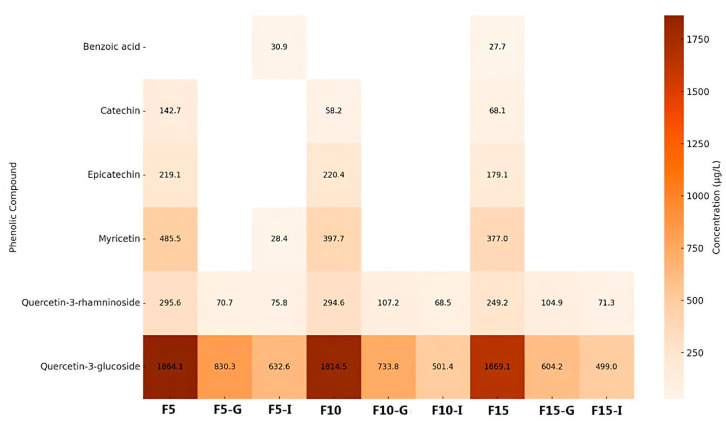
Concentrations of phenolic compounds detected in fruit infusions with steeping times of 5, 10, and 15 min, and their respective gastric and intestinal in vitro digestions. Note: The samples were labeled as follows: F5, F10, and F15 represent fruit infusions with resting times of 5, 10, and 15 min, respectively; F5-G, F10-G, and F15-G correspond to fruit infusions with resting times of 5, 10, and 15 min, respectively, subjected to gastric digestion, while F5-I, F10-I, and F15-I refer to those subjected to intestinal digestion.

Phenolic compounds and other secondary plant metabolites represent a major class of bioactive molecules studied for both research and commercial applications [28]. Therefore, characterizing their phenolic profile is essential to elucidate potential bioactivities. Figure 1 and Figure 2 illustrate the phenolic composition of leaf and fruit infusions of *C. xanthocarpa*, respectively, before and after in vitro gastrointestinal digestion.

Figure 1 illustrates the concentrations of phenolic compounds detected in leaf infusions prepared with steeping times of 5, 10, and 15 min, along with their corresponding gastric and intestinal digestion phases. A total of twelve phenolic compounds, including phenolic acids and flavonoids, were identified across all digestion stages. Chlorogenic acid and kaempferol-3-O-β-rutinoside consistently exhibited the highest concentrations, suggesting greater chemical stability during the digestive process. Quercetin-3-glucoside-7-glucuronide also exhibited elevated concentrations, particularly in the intestinal phase and in infusions with longer steeping times. In contrast, compounds such as epicatechin and hesperidin showed marked reductions following digestion, indicating low digestive stability. Benzoic acid and ferulic acid were generally found at concentrations below the quantification limit in most treatments. Overall, despite reductions, leaf infusions retained significant concentrations of phenolic compounds post-digestion, underscoring their potential bioavailability. Increasing the steeping time generally enhanced the extraction of compounds such as chlorogenic acid and kaempferol-3-O-β-rutinoside, particularly in L15, likely due to increased solubilization facilitated by extended heat exposure. However, this trend was not consistent across all compounds. Other phenolics, including epicatechin and hesperidin, showed a decrease or fluctuation in concentration with prolonged steeping. This behavior may be attributed to thermal degradation, oxidative reactions, or structural transformation of sensitive compounds during extended exposure to high temperatures and oxygen. In some cases, degradation products may not be detected by the analytical method used, which targets specific parent compounds. Therefore, longer steeping times may promote extraction for certain stable phenolics while simultaneously reducing the recoverable content of more labile molecules.

Figure 2 displays the concentrations of phenolic compounds identified in fruit infusions subjected to the same experimental conditions. A total of six phenolic compounds were detected—one phenolic acid and five flavonoids. Unlike the leaf infusions, fruit infusions showed no appreciable increase in phenolic concentrations during digestion. Quercetin-3-glucoside was the most abundant compound across all phases, suggesting high resistance to digestive conditions and favorable bioaccessibility. Myricetin and quercetin-3-rhamnoside were also present in relevant concentrations, though to a lesser extent. Conversely, catechin and epicatechin decreased substantially after digestion, reflecting their susceptibility to degradation. Benzoic acid was present in low concentrations throughout. Steeping time had a variable influence on phenolic content. While longer extraction (F15) enhanced the concentrations of some compounds, others, like catechin and epicatechin, declined, suggesting degradation over time.

A comparative analysis of the two heat maps (Figure 1 and Figure 2) reveals distinct and informative patterns regarding the stability and distribution of phenolic compounds in *C. xanthocarpa* infusions. In both leaf and fruit samples, quercetin and its derivatives demonstrated notable resistance to the digestive process, maintaining elevated concentrations after both gastric and intestinal digestion. This suggests that these flavonols possess intrinsic structural stability, regardless of botanical origin. However, qualitative and quantitative differences were observed between the sources. Fruit infusions exhibited higher concentrations of quercetin-3-glucoside, whereas leaf infusions were richer in chlorogenic acid and kaempferol-3-O-β-rutinoside. These differences likely reflect variations in tissue composition and phenolic metabolism between plant organs. In both matrices, digestion generally led to a reduction in phenolic concentrations, although the extent of degradation varied significantly among compounds. This highlights the heterogeneous stability of phenolics under digestive conditions and the compound-specific nature of bioaccessibility. Regarding the influence of steeping time, longer infusion durations generally promoted greater phenolic extraction, particularly for stable compounds such as chlorogenic acid. Nevertheless, this effect was not universal.

Inconsistencies in the concentration profiles of certain compounds, such as epicatechin and catechin, were observed across different extraction times. For example, epicatechin concentration decreased from L5 to L10 but increased again in L15. This fluctuation cannot be solely attributed to thermal degradation or oxidative loss. Evidence suggests that catechins can undergo oxidation, polymerization, or reversible binding with other matrix components under thermal conditions, influencing their measured concentrations [29,30,31,32]. Wang et al. [33] demonstrated that catechins and their oxidative polymers exhibit variable antioxidant activity and stability depending on temperature and oxygen exposure. Xu et al. [34] also reported that the quantification of flavanols like epicatechin may be influenced by their association with other compounds present in the matrix or by pH-induced changes in solubility. These factors can explain nonlinear extraction profiles, particularly in complex botanical infusions. The reduction in phenolic content during digestion is largely attributed to the pH sensitivity and enzymatic hydrolysis of these compounds [29]. The gastric environment, characterized by a pH of 1.5–3.5, can promote the hydrolysis or decomposition of labile phenolics. However, certain compounds demonstrate resilience under such conditions. For instance, chlorogenic acid, an ester of caffeic and quinic acids, possesses multiple hydroxyl groups that likely enhance its acid stability. Similarly, epicatechin and hesperidin exhibit relatively stable structures, supported by their hydroxyl and glycosidic substitutions, respectively. Kaempferol-3-O-β-rutinoside, with its glycoside moiety, and quercetin-3-glucoside (isoquercitrin), a glycosylated flavonol, also display structural features conducive to stability in acidic environments [30,31,32,33,34,35,36]. Peanparkdee et al. [37], investigating rice bran extracts, reported significant post-digestive reductions in various phenolic acids (e.g., sinapic, protocatechuic, vanillic) and flavonoids (e.g., myricetin, rutin), reinforcing the notion that most phenolics exhibit limited stability under gastrointestinal conditions. On the other hand, Bouayed et al. [38], studying apples, observed a notable increase in phenolic content during the intestinal phase, with over 10% more total phenolics and flavonoids compared to the gastric phase. This rise may be attributed to extended digestion time (exceeding 2 h) and the action of intestinal enzymes, such as pancreatic lipase, which may facilitate the release of matrix-bound phenolics.

These findings emphasize the dynamic nature of phenolic bioaccessibility, which is modulated by multiple factors, including compound structure, food matrix, pH shifts, and enzymatic activity. Phenolics often undergo structural modifications during digestion, forming glycosylated, esterified, or polymerized intermediates, which are further hydrolyzed or transformed under acidic and alkaline conditions [39]. Such transformations ultimately influence the fraction of phenolics that remain bioaccessible and potentially bioavailable.

### 3.2. Bioaccessibility Index of Phenolic Compounds of the Infusions

**Figure 3:**
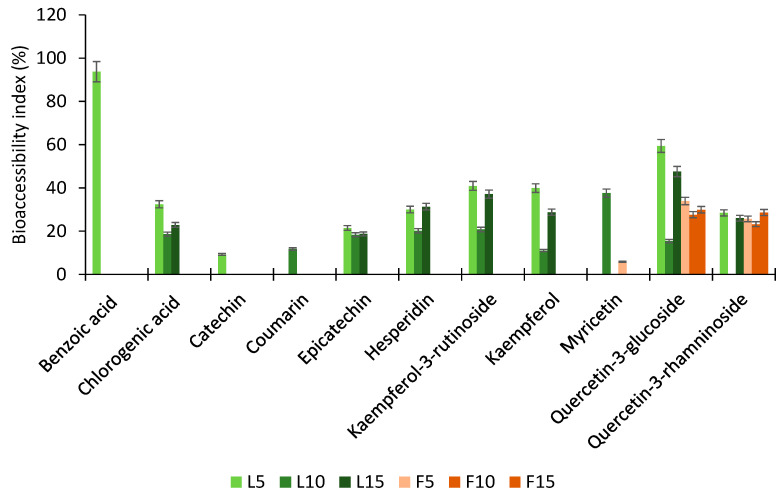
Bioaccessibility index (%) of phenolic compounds after the in vitro gastrointestinal digestion process of *Campomanesia xanthocarpa* leaf and fruit infusions.

To assess the stability and transformation of phenolic compounds following simulated gastrointestinal digestion, the *BI* was calculated for each compound after the complete digestion process (Figure 3).

A range of phenolic acids (e.g., benzoic acid, chlorogenic acid) and flavonoids (e.g., catechin, coumarin, epicatechin, hesperidin, kaempferol-3-O-β-rutinoside, kaempferol, myricetin, quercetin-3-glucoside, and quercetin-3-rhamnoside) was evaluated. Among all compounds, benzoic acid in L5 exhibited the highest *BI*, reaching approximately 93.70%, indicating strong resistance to degradation during digestion. Chlorogenic acid also demonstrated moderate bioaccessibility, with values of 32.54% in L5, 18.60% in L10, and 22.88% in L15, showing slight variability across infusion times. Catechin showed relatively low bioaccessibility, with a *BI* of 9.20% in L5, suggesting poor digestive stability. In contrast, coumarin and myricetin in L10 presented *BI*s of 11.76% and 37.59%, respectively. Quercetin-3-rhamnoside demonstrated moderate stability, with BIs of 28.48% in L5 and 26.00% in L15. Other compounds, including epicatechin, hesperidin, kaempferol-3-O-β-rutinoside, kaempferol, and quercetin-3-glucoside, maintained detectable and consistent bioaccessibility across all steeping durations. In the fruit infusions, myricetin exhibited a low BI of 5.85% in F5, while quercetin-3-glucoside had the highest *BI* among fruit-derived compounds, with values of 33.94% in F5, 27.63% in F10, and 29.90% in F15, indicating considerable resistance to digestive conditions. Likewise, quercetin-3-rhamnoside maintained relevant bioaccessibility across steeping times, with *BI*s of 25.64% in F5, 23.25% in F10, and 28.61% in F15. Overall, these findings suggest that glycosylated flavonoids, such as quercetin derivatives, tend to exhibit higher digestive stability compared to non-glycosylated phenolics. The variation in *BI* among different compounds also reinforces the influence of molecular structure and extraction time on phenolic bioaccessibility.

Dantas et al. [40] investigated the bioaccessibility of frozen pulps from Brazilian native fruits, including açaí, cupuaçu, blackberry, blueberry, jabuticaba, raspberry, cajá, and soursop. The study revealed a wide range of bioaccessibility levels across different phenolic compounds and fruit matrices. For example, epicatechin in açaí pulp showed a bioaccessibility of 32.99%, while quercetin-3-glucoside exhibited highly variable values—122.01% in blackberry and 13.94% in blueberry. Hesperidin presented bioaccessibility levels of 13.91% in cajá and 28.01% in açaí, highlighting the influence of both the compound and the matrix on digestive stability. In fruits, phenolic acids may occur in the form of free glycosides or soluble esters, which contribute significantly to their antioxidant potential. Thus, it is essential to evaluate the bioaccessibility of phenolics in both whole fruits and their derivatives, as matrix composition can markedly affect compound release and transformation [41]. Within the phenolic acid class, chlorogenic acid was also evaluated by Dantas et al. [40], showing bioaccessibility levels of 18.28% in açaí and 15.65% in blueberry. The decrease in chlorogenic acid concentration during digestion may be attributed to partial hydrolysis into derivatives such as caffeoylquinic acid and dimethoxycinnamoylquinic acid [42]. Similarly, Yu et al. [42] reported bioaccessibility values for plum phenolic extracts, including chlorogenic acid (23.11%), epicatechin (26.66%), and isoquercitrin (12.40%). The moderate bioaccessibility of epicatechin may be linked to the degradation of procyanidin B2, a dimer of epicatechin. During gastrointestinal digestion, procyanidin B2 can be depolymerized into monomeric units, including epicatechin, thereby increasing its measurable concentration in the intestinal phase [43]. The changes observed in the *BI* of phenolic compounds after in vitro gastrointestinal digestion can be attributed to the complex interactions and transformations these compounds undergo during digestion. Phenolic acids, such as benzoic acid and chlorogenic acid, are known for their relative stability in acidic environments like the gastric phase, which can explain their detectable and consistent *BI* levels. Chlorogenic acid, despite partial degradation, forms derivatives with comparable bioactivity, contributing to its stability across various extraction times [44,45]. Flavonoids, including catechin, epicatechin, hesperidin, and quercetin derivatives, are often influenced by their glycosylation patterns. Glycosylated flavonoids, such as quercetin-3-glucoside and quercetin-3-rhamnoside, tend to be more stable during digestion due to the protective effect of sugar moieties, which can be hydrolyzed to release aglycones during intestinal digestion. This process enhances their bioavailability, which is reflected in the significant *BI* levels observed [45,46]. Compounds like myricetin and kaempferol derivatives show variability in BI, likely due to their sensitivity to pH changes and enzymatic hydrolysis. Myricetin’s relatively high BI in certain conditions may result from its resistance to degradation and effective release during intestinal digestion. Conversely, the lower *BI* of catechin and coumarin may stem from their susceptibility to oxidation or interaction with digestive enzymes, leading to reduced stability [44]. The observed variations between leaf and fruit infusions could be linked to intrinsic matrix effects, such as the presence of other bioactive compounds, dietary fiber, or proteins, which may modulate the release and stability of phenolic compounds throughout digestion. Additionally, extraction time influences the initial phenolic yield, thereby impacting their subsequent *BI*. In the present study, infusion times (5, 10, and 15 min) were selected based on preliminary trials and designed to emulate customary household preparation practices. This methodological choice aimed to enhance the translational value of the findings by reflecting realistic dietary scenarios rather than maximizing compound recovery under optimized laboratory conditions. These findings underscore the importance of both phenolic structure and food matrix composition in determining digestive stability and bioavailability.

### 3.3. TPC and TAC of the Infusions

Table 2 presents the results for *TPC* and total *TAC* of *C. xanthocarpa* leaf and fruit infusions, both before and after in vitro gastrointestinal digestion. Phenolic compounds, which are widely distributed in plants, are recognized as potent antioxidants. Phenolic acids such as chlorogenic, gallic, caffeic, and ellagic acids exert antioxidant activity primarily through the donation of hydrogen atoms from hydroxyl groups on their aromatic rings. Additional mechanisms include electron transfer and quenching of singlet oxygen species [47]. Natural flavonoids such as kaempferol, quercetin, and myricetin, commonly found in various plant-based foods, also exhibit robust antioxidant effects [48,49,50].

As expected, the undigested infusions exhibited the highest *TPC*, with the exception of sample L10. Gastrointestinal digestion significantly influenced the phenolic content of the samples. In leaf infusions, reductions in *TPC* were observed after the gastric phase: 36.38% (L5-G), 2.46% (L10-G), and 2.39% (L15-G). During the intestinal phase, reductions of 57.26% (L5-I) and 47.00% (L15-I) were observed, whereas L10-I exhibited a 29.77% increase in *TPC*. This increase may be attributed to the action of intestinal enzymes, which facilitate the release of phenolic compounds previously bound to the plant matrix [51]. For fruit infusions, digestion also led to significant *TPC* reductions: 57.80% (F5-G), 25.05% (F10-G), and 15.03% (F15-G) during the gastric phase and 61.42% (F5-I), 10.52% (F10-I), and 35.51% (F15-I) during the intestinal phase. On average, leaf infusions exhibited *TPC* reductions of 13.75% (gastric) and 24.83% (intestinal), while fruit infusions showed higher average reductions of 32.63% (gastric) and 35.56% (intestinal). These results indicate greater phenolic stability in leaf infusions under simulated gastrointestinal conditions. These findings are consistent with previous literature. Celep et al. [52] reported an approximately 57% reduction in *TPC* during intestinal digestion. Similarly, Kashyap et al. [53] observed a 49.79% decrease in phenolic content in the digested extracts of Meghalayan cherry. Farias et al. [54] found 35% and 50% reductions in the *TPC* of uvaia during the gastric and intestinal phases, respectively. According to Pavan et al. [55], the reduction in *TPC* during digestion is likely due to the instability of smaller phenolic molecules released through hydrolysis at higher pH values, particularly in the intestinal environment.

The *TAC* of *C. xanthocarpa* infusions, measured before and after in vitro digestion, exhibited a notable reduction across all samples. In the intestinal phase, *TAC* values for the leaf infusions decreased by 51.65% (L5-I), 58.87% (L10-I), and 52.09% (L15-I). For the fruit infusions, the reductions were even more pronounced—61.19% (F5-I), 64.55% (F10-I), and 61.16% (F15-I)—for the corresponding steeping times (5, 10, and 15 min). These findings are supported by previous studies. Farias et al. [54] reported a 21% decrease in the antioxidant potential of digested uvaia extracts, while Dacoreggio et al. [23] observed an 18.75% reduction during the gastric phase. Similarly, Ma et al. [56] noted a progressive decline in antioxidant activity during the digestion of pea peel samples, suggesting a consistent pattern across plant-based matrices. This reduction in *TAC* may be attributed to the varied structural forms of phenolic compounds within food matrices—namely, free, conjugated, or bound phenolics—which influence their release and reactivity under digestive conditions [57]. Furthermore, enzymatic activity (e.g., pepsin, pancreatin) and pH transitions during gastrointestinal simulation likely contribute to the breakdown or transformation of antioxidant compounds, thereby altering their capacity to scavenge free radicals. Despite the post-digestion decline, leaf infusions consistently exhibited higher antioxidant capacity than fruit infusions prior to digestion, reflecting a greater initial concentration of bioactive compounds. Even after intestinal digestion, leaf infusions maintained a superior antioxidant profile, suggesting that their phenolic constituents are not only more abundant but potentially more stable or reactive. The observed decrease in *TAC* reinforces the complexity of antioxidant mechanisms and highlights the importance of complementary assays for a comprehensive understanding of how digestion affects the antioxidant potential of plant-derived foods.

### 3.4. Inhibition of Starch-Digesting Enzymes by Infusions

**Figure 4:**
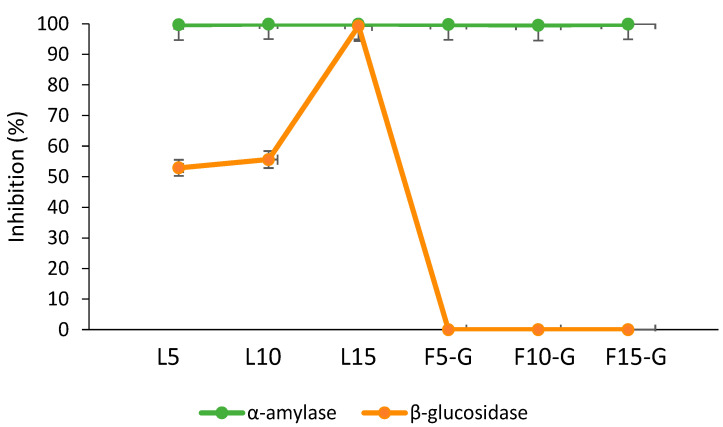
Percentage inhibition of α-amylase and β-glucosidase by *C. xanthocarpa* leaf and fruit infusions prepared at different extraction times (5, 10, and 15 min). Note: Samples used for β-glucosidase inhibition were previously subjected to simulated gastric digestion, reflecting the physiological digestive progression to the small intestine.

Figure 4 illustrates the percentage inhibition of α-amylase and β-glucosidase by *C. xanthocarpa* leaf and fruit infusions prepared with steeping times of 5, 10, and 15 min. All samples exhibited marked inhibition of α-amylase, with values ranging from 99.54% to 99.91%. In contrast, β-glucosidase inhibition was observed exclusively in the leaf infusions, with inhibition increasing progressively with extraction time—from 52.9% at 5 min (L5) to 99.3% at 15 min (L15). Since β-glucosidase activity primarily occurs in the small intestine, the inhibition assay was performed on samples that had undergone simulated gastric digestion, thereby reflecting the physiological sequence of the digestive process. The strong inhibition of α-amylase by *C. xanthocarpa* infusions suggests the presence of potent bioactive compounds capable of modulating early steps in carbohydrate digestion. These findings are consistent with previous studies indicating that phenolic compounds can act as natural α-amylase inhibitors, representing potential alternatives to synthetic drugs for controlling postprandial glycemia and managing type 2 diabetes [58]. Flavonoids, in particular, have been shown to modulate carbohydrate metabolism by reducing starch digestibility and by inhibiting glucose transporters such as SGLT-1 in the intestinal epithelium, thereby enhancing glycemic control [59]. Although both leaf and fruit infusions demonstrated high α-amylase inhibition, leaf infusions (99.67–99.91%) outperformed fruit infusions (99.54–99.84%), likely due to their higher concentrations of specific phenolics such as quercetin, gallic acid, and chlorogenic acid, as evidenced in the phenolic profile [60,61].

Supporting this, Etgeton et al. [62] reported concentration-dependent α-amylase inhibition by aqueous fruit extracts of *C. xanthocarpa*, with inhibition ranging from 20% to 77%, highlighting the plant’s potential as a natural source of enzyme inhibitors. Similar results have been reported for other species; for instance, Mushtaq et al. [63] observed significant inhibitory effects on both α-amylase (70%) and β-glucosidase (65%) in *Calligonum polygonoides*, reinforcing the functional relevance of flavonoids and phenolic acids. With regard to β-glucosidase, inhibition was restricted to leaf infusions and increased with steeping time, suggesting a time-dependent release of inhibitory phytochemicals. Since these samples were analyzed after simulated gastric digestion, this trend may also reflect the greater digestive stability of flavonoids extracted at longer steeping times, which remained active post-digestion. Flavonoids such as quercetin and kaempferol, which are abundant in leaves of the Myrtaceae family, have demonstrated high affinity for the active site of β-glucosidase [64]. Thus, the inhibitory activity observed in *C. xanthocarpa* leaves can be attributed to the presence of these compounds. Conversely, no β-glucosidase inhibition was detected in the fruit infusions, regardless of the extraction time (0% inhibition). This absence of activity suggests that the responsible bioactives are either absent or present at insufficient concentrations in the fruit matrix. This pattern is consistent with findings from other botanical studies showing that leaves typically accumulate higher levels of enzyme-inhibiting phenolics, while fruits tend to be richer in sugars and non-inhibitory constituents. For example, Wojdyło et al. [65] compared the leaves and fruits of apple, pear, and quince, reporting that leaf extracts had higher phenolic content and greater inhibitory activity against α-amylase and α-glucosidase than fruit extracts. In particular, quince leaves were classified as potent α-amylase inhibitors. Similarly, Spínola et al. [66] demonstrated that the leaves of *Elaeagnus umbellata* and *Sambucus lanceolata* exhibited stronger glucosidase inhibition than their fruits. Furthermore, Gong et al. [67] emphasized that flavonoids possess superior glucosidase inhibitory capacity compared to phenolic acids, likely due to additional hydroxyl groups on the flavonoid backbone, which enhance enzyme binding. In line with this, Phan et al. [68] demonstrated that flavonoids such as baohuoside I, kaempferol, and quercetin inhibited yeast glucosidase by 82.6%, 95.1%, and 94.7%, respectively, while compounds like icariin and epimedins showed inhibition below 5%. Together, these findings underscore the therapeutic potential of *C. xanthocarpa*, particularly its leaves, as a natural source of bioactive compounds capable of modulating key digestive enzymes involved in carbohydrate metabolism.

### 3.5. In Vivo Evaluation of Biomarkers

The data for fasting glucose, serum fructosamine, and amylase activity are presented in Table 3. The administration of dry baked biscuits containing *C. xanthocarpa* leaf infusion did not result in statistically significant differences in fasting glucose levels (*p* = 0.19) or serum amylase activity (*p* = 0.84) compared to the control group. However, a significant treatment-by-time interaction was observed for fructosamine levels (*p* = 0.05), suggesting a time-dependent modulation of intermediate glycemic control. Fructosamine reflects the non-enzymatic glycation of serum proteins, primarily albumin, and serves as a marker of average glycemia over the preceding two to three weeks. In contrast to fasting glucose, which is subject to rapid physiological fluctuations, fructosamine offers a more stable and integrative assessment of glycemic status [69,70].

The elevated fructosamine levels detected at specific time points in the treatment group—especially on day 11—may reflect a metabolic response to the phenolic compounds present in the *C. xanthocarpa* infusion added to the biscuits. These compounds can influence glucose metabolism through multiple mechanisms, including the modulation of intestinal glucose absorption, inhibition of carbohydrate-digesting enzymes, and interference with hepatic glucose production or insulin signaling pathways [71,72]. Although the overall mean glucose levels remained unaffected, the time-dependent variation in fructosamine levels suggests that the phenolic-enriched biscuits may exert effects through cumulative or delayed physiological responses rather than through immediate glycemic shifts. These physiological findings are consistent with the results of the in vitro enzymatic assays, in which the same *C. xanthocarpa* infusion used in biscuit formulation significantly inhibited both salivary α-amylase and β-glucosidase activities. The inhibition of these key enzymes involved in starch digestion may delay carbohydrate breakdown and glucose release into the bloodstream, thereby contributing to the observed modulation of fructosamine levels over time [73].

It is also important to consider that phenolic compounds can modulate protein glycation processes beyond their role in postprandial glucose control. Studies have demonstrated that flavonoids such as quercetin, rutin, and caffeoyl malic acid can significantly reduce the formation of advanced glycation end-products (AGEs) and protein oxidation markers in vitro, even in the presence of elevated glucose concentrations. These effects occur through multiple mechanisms, including dicarbonyl scavenging, antioxidant activity, and stabilization of protein structures during glycation [74].

Although this mechanism did not result in a significant reduction in fasting glucose, it may partially explain the fluctuations observed in intermediate glycemic markers, supporting a subtle but biologically relevant regulatory role for the phenolic compounds delivered through the dry biscuits. Additionally, the interaction between the food matrix and phenolic compounds may have influenced the bioaccessibility and functionality of these bioactives in vivo. The biscuit matrix, due to its dry and compact nature, could modulate the release profile of phenolics during digestion, potentially slowing their degradation and enabling more sustained physiological effects [13,75]. Such matrix-related interactions are crucial for understanding how bioactive compounds behave under real dietary conditions and may help explain the time-dependent effects observed in fructosamine levels.

Although direct markers of oxidative stress were not evaluated in vivo, the observed modulation of serum fructosamine may also reflect, in part, the antiglycation or antioxidant potential of the phenolic compounds retained after digestion. Given that advanced glycation processes are closely linked to oxidative stress, the reduction or stabilization of protein glycation markers could suggest a systemic effect of bioaccessible phenolics beyond their role in glycemic control [76,77,78]. This hypothesis is supported by the strong in vitro antioxidant activity observed, especially in the leaf infusions.

The delivery of phenolic compounds through a complex food matrix such as dry biscuits may have influenced their release profile, contributing to the delayed modulation of glycemic markers like fructosamine. Phenolic–protein interactions—particularly those formed during thermal processing—can act as controlled-release systems, protecting bioactive compounds from early degradation and ensuring their gradual release throughout the gastrointestinal tract [79]. Such interactions may also explain why no immediate reduction in fasting glucose was observed, while a significant time-dependent effect emerged in fructosamine levels.

In this context, the proteins present in the biscuit formulation, including quinoa-derived proteins and gelatin, are likely to interact with polyphenols through hydrogen bonding or covalent attachments [15]. These interactions may not only protect phenolics from enzymatic hydrolysis but also influence protein digestibility and intestinal absorption dynamics. Studies have shown that such complexes can modify the tertiary structure of proteins, altering their hydrolysis rate and possibly enhancing the release of phenolics at more distal sites of digestion [80,81].

This finding also points to the relevance of integrative experimental approaches combining in vitro biochemical assays and physiological validation. Future studies should explore additional metabolic markers, including oxidative stress parameters, to further clarify the mechanisms by which *C. xanthocarpa* phenolics influence glycemic homeostasis. Furthermore, the apparent dissociation between fructosamine and glucose responses may indicate a compensatory adjustment in insulin sensitivity or peripheral glucose utilization, which could be further explored in studies involving insulin quantification, glucose tolerance tests, or gene expression analysis of glucose transporters and related metabolic regulators [82,83,84]. Overall, the interaction between phenolics and the food matrix should not be regarded merely as a barrier to absorption, but rather as a functional mechanism that can modulate bioactive compound behavior in vivo.

## 4. Conclusions

This study provides an integrated assessment of the functional potential of *C. xanthocarpa* infusions through phytochemical profiling, in vitro digestion, enzyme inhibition assays, and in vivo evaluation of glycemic markers. Leaf infusions showed higher total phenolic content, antioxidant capacity, and enzyme inhibitory activity compared to fruit infusions. Several phenolic compounds, particularly glycosylated flavonoids, remained bioaccessible after simulated digestion, and their concentrations were influenced by steeping time and the food matrix. In vivo, the administration of biscuits enriched with leaf infusion resulted in a time-dependent modulation of serum fructosamine, suggesting a physiological effect on glycemic regulation. These findings contribute to the understanding of *C. xanthocarpa* as a potential functional ingredient with relevance to metabolic health, supporting its future application in functional food development. Nonetheless, the study has limitations, including its short-term duration, use of healthy animals, and the absence of additional metabolic and inflammatory markers. These factors should be addressed in future research to better understand the physiological relevance and translational potential of *C. xanthocarpa* phenolics.

## Figures and Tables

**Table 1:** Ingredient composition of control and treatment dry baked biscuit formulations (% *w*/*w*), with representative image of the final product.

Ingredient	Control (%)	Treatment (%)	Visual Aspect
*C. xanthocarpa* leaf extract *	–	0.40	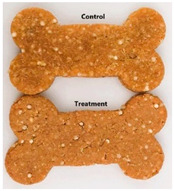
Industrialized sesame biscuit **	29.89	29.49	
Quinoa	12.64	12.64	
Potassium sorbate	0.16	0.16	
Bacon flavoring	0.42	0.42	
Unflavored, colorless gelatin	4.21	4.21	
Distilled water	52.68	52.68	

Note: * *C. xanthocarpa* leaf extract corresponds to lyophilized leaf infusion (L15), and the 0.4% represents its proportion in the formulation on a weight/weight basis. ** The sesame biscuit used in both formulations was obtained from the brand Orquídea (Caxias do Sul, Brazil).

**Table 2:** Total phenolic content (*TPC*) and total antioxidant capacity (*TAC*) of *C. xanthocarpa* leaf and fruit infusions before and after simulated in vitro gastrointestinal digestion.

Sample	*TPC* (mg de EGA. g^−1^)			*TAC* (μM)	
	Infusion	Gastric Digestion	Intestinal Digestion	Infusion	Intestinal Digestion
**L5**	204.20 ±8.47 ^aA^	129.90 ± 44.77 ^aB^	87.26 ± 25.47 ^aB^	3028.33 ± 223.6 ^aA^	1470.00 ± 51.64 ^aB^
**L10**	94.85 ± 9.81 ^cA^	92.51 ± 15.59 ^bA^	123.10 ± 45.08 ^aA^	2491.67 ± 85.66 ^bA^	1025.00 ± 162.7 ^bB^
**L15**	154.20 ± 15.65 ^bA^	150.50 ± 5.97 ^aA^	81.72 ± 37.78 ^aB^	2931.67 ± 83.14 ^aA^	1405.00 ±15.83 ^aB^
**F5**	129.90 ± 49.00 ^bA^	54.83 ± 11.90 ^cB^	50.11 ± 2.97 ^cB^	1288.33 ± 75.05 ^cA^	500.00 ± 27.86 ^cB^
**F10**	92.52 ± 32.05 ^cA^	69.34 ± 4.70 ^cA^	82.78 ± 14.94 ^aA^	1316.67 ± 113.9 ^cA^	466.67 ± 27.95 ^cB^
**F15**	107.80 ± 5.21 ^cA^	91.59 ± 36.64 ^bA^	69.52 ± 3.21 ^bB^	1321.67 ± 62.30 ^cA^	513.33 ± 62.35 ^cB^

Note: L5, L10, and L15 represent leaf infusions with resting times of 5, 10, and 15 min, respectively; F5, F10, and F15 represent fruit infusions with resting times of 5, 10, and 15 min, respectively. Values followed by different lowercase letters in the same column differ significantly (*p* < 0.05). Different uppercase letters in the same row indicate significant differences (*p* < 0.05).

**Table 3:** Mean values (±SEM) of fasting glucose, serum fructosamine, and amylase activity in control and treatment groups over the experimental period.

Variables	Control	Treatment	SEM	*p*: Treatment	*p*: Treatment × Day
Amylase (U/L)				0.84	0.92
Day 1	1883	1796	71.3		
Day 11	1939	1935	70.8		
Day 18	1863	1754	74.1		
Day 25	1769	1704	66.2		
Day 32	1854	1897	70.1		
Mean	1856	1822	70.6		
Glucose (mg/dL)				0.19	0.22
Day 1	104	103	2.89		
Day 11	100	112	3.12		
Day 18	106	108	3.12		
Day 25	103	111	3.09		
Day 32	102	107	2.97		
Mean	102.75	109.5	3.01		
Fructosamine (µmol/L)				0.39	0.05
Day 1	136	133	3.14		
Day 11	132 ^b^	150 ^a^	3.09		
Day 18	131	142	2.98		
Day 25	137	149	3.12		
Day 32	146	147	3.08		
Mean	136	147	3.11		

Note: Different letters indicate significant differences (*p* ≤ 0.05) in the treatment × time interaction.

## Data Availability

The data supporting the findings of this study are available from the corresponding author upon reasonable request.
